# Computer analysis of the sensory qualities of red wines as a method to optimize their blend formulation

**DOI:** 10.1016/j.heliyon.2019.e01602

**Published:** 2019-05-10

**Authors:** A.A. Khalafyan, Z.A. Temerdashev, Yu. F. Yakuba, T.I. Guguchkina

**Affiliations:** a Kuban State University, 149, Stavropolskaya St., Krasnodar, 350040, Russia; b North Caucasian Federal Research Center of Horticulture, Viticulture, Wine-making, 39, 40-let Pobedy St., Krasnodar, 35090, Russia

**Keywords:** Food technology, Food safety, Food analysis, Food science, Analytical chemistry

## Abstract

Three high quality red wines – Merlot, Cabernet Sauvignon and Pinot Noir – were used for development of an optimized formulation of a new blended wine called *Zvezda Kubani* (“The Star of Kuban”). The experimental plan was implemented with the mixture designs and triangular surfaces module in the STATISTICA package. According to the experimental plan, we made and studied 31 variants of wines, including 3 monovariants, 3 mixtures of 2 wines and 25 mixtures of 3 wines. In addition, highly qualified specialists have studied the changes in the mixtures according to the results of a sensory assessment to model the connection between the sensory perception of wine mixtures and the new blended wine formulation. As a result, we developed a mathematically proved formulation of a new blended wine, *Zvezda Kubani*, containing 48% Merlot, 35% Cabernet Sauvignon and 17% Pinot Noir. The experimental verification of the suggested composition of the blend proved to be a strong indicator of the experts' sensory assessment.

## 1 Introduction

The growth of blended wines is currently being observed in traditional wine-producing countries. From the data [1], the actual sales figures for this category of wines indicate a significant coverage of the wine market. To improve quality and achieve the desired sensory perception, many researchers are experimenting with blending different base wines in certain proportions [2, 3, 4, 5, 6].

The labour intensity of blending is due to the great number of possible variations of base wines proportions; mathematically, their number cannot be measured by a finite number; there are infinite variations. However, sensory wine qualities are the result of the influence of numerous compounds with different perception thresholds that determine how their consumer properties interact with each other [7]. Currently, many winemakers use mathematical and statistical methods, including those implemented in application packages, to compose blends with the best sensory qualities.

Hopfer H. et al. [2] produced 11 mixtures of two wines and four mixtures of three wines to study the changes in their sensory perception using descriptive analysis of three varietal wines (Cabernet Sauvignon, Merlot and Cabernet Frank). In addition, they carried out chemical analyses to assess the possible correspondence of sensory perception of wine mixtures to the measured chemical parameters. Using canonical discriminant analysis (CDA), multi-dimensional and one-dimensional variance analysis (MANOVA and ANOVA), and correlation analysis for each type of wine and the mixture components, they analysed the taste, olfactory and visual indicators, sampling assessments and chemical analysis results. The comparison of all datasets showed that the overall results of sensory and chemical analyses were in satisfactory agreement, but the most representative were the results obtained using descriptive analysis. Vismara P. et al. [3] described a decision support system to create a wine blend by mixing base wines while optimizing the content of the chemical compounds that influence the sensory characteristics of wine. The optimization model was based on disjunctive constraints that can be constructed by Boolean variables. Dooley L. et al. [5] used experiment planning, namely, the analysis of mixtures making up blending wines (Cabernet Sauvignon, Merlot and Zinfandel), to optimize the wine composition to satisfy consumers' tastes. A linear model of simplex-centroid plans for mixtures designs was used. The main correlations were revealed between compositional and colour data in comparison with sensory or consumer assessments. Dooley L. et al., in another publication [6], used the mixture designs and triangular surfaces method to create a blend of Cabernet Sauvignon, Merlot and Zinfandel wines. After 12 months of blending storage, colour, chemical composition and anthocyanin content were examined. The sensory assessment showed that blending light wine with saturated wine positively influenced its perception by the consumer. Ghanem E. et al. [8] examined 15 wine mixtures based on Cabernet Sauvignon, Merlot and Cabernet Frank to create a sensory assessment prediction model for two- and three-component mixtures using discriminant, correlation and regression analyses. The results of the sensory evaluation and the content of tannins in the test samples were chosen as the criteria for blend quality. Cross-checking the results allowed a quantitative prognostic model of red wine composition to be proposed. Applying various methods of making blends based on three varietal wines — Muscat Bailey A, Gerbongand and Campbell — using the method of numerical and graphical optimization, the authors [4] suggested that the composition of 11.1% Gerbong, 48.9% Campbell and 40.0% Muscat Bailey A had the best sensory result. Wine quality criteria in this work were taste preferences, colour and total content of polyphenols.

Numerous aspects determine the quality of wines, such as their sensory properties or micro-element compositions, which makes them difficult to objectively assess. Therefore, work devoted to the use of probability and statistical models implemented in statistical packages to assess the quality of wine products [9, 10, 11, 12, 13] are typical. Zoecklein B.W. et al. [9] assessed the ability of two electron nose systems (conductive polymer and surface acoustic wave) to differentiate the volatile substances of Cabernet Frank and Merlot wines after spraying the vineyard with a 5% aqueous solution of ethanol using CDA, ANOVA and principal component analysis (PCA). Hopfer H. et al. [10] presented data on the chemical and sensory analysis of 27 different samples of Californian Cabernet Sauvignon wine of different quality categories based on the relationships between wine characteristics and their sensory assessment, analysed using ANOVA and PCA. Cadota Y. et al. [11] performed a comparative analysis of the sensory assessment of 24 Cabernet Frank wine samples using two groups of taster, one group for those who did not work in the wine industry and the other for wine specialists, using a wide range of statistical procedures (quantitative descriptive analysis, ANOVA, PCA and correlation analysis).

In other studies [12, 13, 14, 15, 16], various mathematical optimization methods, such as experimental planning, regression, nonlinear models realized through neural networks and linear programming models, were applied to study blending procedures. From some papers [17, 18], we studied different approaches to evaluate wine quality using statistical methods implemented in the STATISTICA package [19]. A wide-range of methods included in this package were considered: multiple regression, CDA, the general linear model, item analysis, table cross tabulation, correspondence analysis, cluster analysis and logit regression. All things considered, the modern methods of statistical data analysis are highly effective in creating blended wines with the best qualities.

This research considers the mixture design and triangular surfaces method implemented with the STATISTICA package to formulate the *Zvezda Kubani* blended wine produced from the varietal wines grown in the Kuban region using their sensory properties as quality criterion.

## 2 Materials and methods

### 2.1 Objects of research

Three varietal wines (2016 harvest), Cabernet Sauvignon (CS), Merlot (M) and Pinot Noir (PN), were used for the experiments. All the grapes for the three wines were grown by the agricultural firm "Yubileinaya," in the Zaporozhskaya village of the Temryuk district, Krasnodar region. These grape varieties have been successfully cultivated for many years in Krasnodar region. The samples of varietal wines made from grapes were provided for research by the producer—the agricultural firm "Yubileinaya." All three samples of wine were dry (residual sugar: CS = 0.3%, M = 0.4%, PN = 0.6%) and the alcohol content ranged from 11.4 v% to 12.3 v% (CS = 11.8 v%, M = 11.4 v%, PN = 12.3 v%). The wines were mixed one week after their delivery to Krasnodar. During this period, 15.2 ± 0.05 dm^3^ of each wine was stored in dark green glass bottles with screw caps at 10 °C until further use. The pH varied from 3.61 to 3.79 (CS = 3.76, M = 3.61, PN = 3.79). The level of dissolved oxygen of the basic varietal wines was measured by immersion of a probe before casking and was below 1 mg/dm^3^ for all wines (CS = 0.47 mg/dm^3^, M = 0.68 mg/dm^3^, PN = 0.39 mg/dm^3^). After mixing, another measurement of dissolved oxygen was made and the oxygen remained below 1 mg/dm^3^—the wine with the highest value, 0.57 mg/dm^3^, was used for blend 7 in Table 1. In total, we analysed 31 blends that included three varietal base wines, 3 wines from two varieties and 25 wines from three varieties with different ratios of each base wine, as shown in Table 1.

**Table 1:** Sensory assessment plan for samples of blended wines.*

Standard Run	3-factor simplex-lattice design (Degree m = 2) (Star Kuban.sta) + interior points and overall centroid. Sum of all mixture components: 100			
	Merlot	Cabernet Sauvignon	Pinot Noir	**DV
1	100.00	0.00	0.00	78.00
2	0.00	100.00	0.00	74.00
3	0.00	0.00	100.00	70.00
4	50.00	50.00	0.00	87.00
5	50.00	0.00	50.00	82.00
6	0.00	50.00	50.00	83.00
7	66.67	16.67	16.67	92.00
8	16.67	66.67	16.67	81.00
9	16.67	16.67	66.67	83.00
10	33.33	33.33	33.33	87.00
11	80.00	10.00	10.00	81.00
12	90.00	5.00	5.00	75.00
13	5.00	90.00	5.00	70.00
14	5.00	5.00	90.00	65.00
15	10.00	80.00	10.00	74.00
16	10.00	10.00	80.00	68.00
17	45.00	45.00	10.00	85.00
18	45.00	10.00	45.00	85.00
19	10.00	45.00	45.00	75.00
20	56.67	21.67	21.67	86.00
21	21.67	56.67	21.67	75.00
22	21.67	21.67	56.67	78.00
23	60.00	20.00	20.00	79.00
24	20.00	60.00	20.00	75.00
25	20.00	20.00	60.00	78.00
26	40.00	40.00	20.00	85.00
27	40.00	20.00	40.00	83.00
28	20.00	40.00	40.00	80.00
29	46.67	26.67	26.67	88.00
30	26.67	46.67	26.67	88.00
31	26.67	26.67	46.67	77.00

*All the tables, inscriptions, and original graphs were built by the STATISTICA package **DV – dependent variable.

### 2.2 Sensory analysis

All experimental research connected with sensory analysis was performed at the Federal Research Centre of Horticulture, Viticulture and Wine-Making (FSC HVW), Krasnodar, Russia. The general descriptive analysis was made for 31 wine samples, including 3 mono-variational, 3 two-component and 25 three-component mixtures of varietal wines (Merlot, Cabernet Sauvignon and Pinot Noir). The sensory assessment procedure involved 15 specialists (9 women and 6 men) in the age ranges of 32–45 (12 people) and 55–66 (3 people). All the participants are considered wine experts, work in the wine industry and have professional experience in the field of sensory analysis. The sensory assessment results of wine quality were expressed on a scale from 50 to 100 by the famous Parker rating system [20]. They calculated the arithmetic mean of the sensory assessment values to the first decimal place. The wines were served in transparent, tulip-shaped wine glasses with a capacity of 220 cm^3^. A sample (50 cm^3^) was poured into each glass and covered with a Petri dish with a diameter of 5.7 cm 30 minutes before the sensory assessment. The tests were conducted in a lit tasting room with a controlled temperature regime. All samples were served at 16–22 °C at tables with white napkins. Experts were forbidden to communicate during the sensory assessment procedure. The intervals between tasting each sample were 2 minutes. Two additional 3-minute intervals between the third, fourth, sixth and seventh samples were used to reduce fatigue. During each break, the experts rinsed their mouths with water. The tasters evaluated each blend in three repetitions during the working week. The experts performed a sensory assessment of the mixtures produced by the formulations shown in Table 1. The percentages of the mixture components for each experiment are indicated in Table 1; the sum of the fractions in each row of the table is equal to 100%.

### 2.3 Statistical analyses

Developing the experimental plan to determine the formulation of blended wine and its analysis were performed using the STATISTICA v.10 (Tibco, USA) package [19]. The mixture designs and triangular surfaces module generated a three-factor simplex vertex plan consisting of 31 experiments.

The dispersion analysis allowed assessment of the adequacy of the linear and quadratic models constructed by the module. Using a Pareto chart, a trace plot and profiles for predicted values and desirability, approximate-optimal values of the quadratic function were found, which allowed the share composition of the wines to be obtained. To compare, MATLAB package MATLAB v.5 (MathWorks, USA) package [21], was also used to find the optimal solution of the quadratic programming problem.

## 3 Results and discussion

### 3.1 Developing an experimental plan and choosing a mathematical model

The choice of the component composition of the blend was determined by the original properties of the basic red wines produced from the same grape varieties in one wine-making enterprise. When setting the start window of the mixture designs and triangular surfaces module, the number of factors was set to 3, the polynomial degree was set to 2 and the augment with interiors & centroid option was selected to develop the three-factor simplex-vertex experimental plan for the blended samples of wines presented in Table 1.

**Fig. 1:**
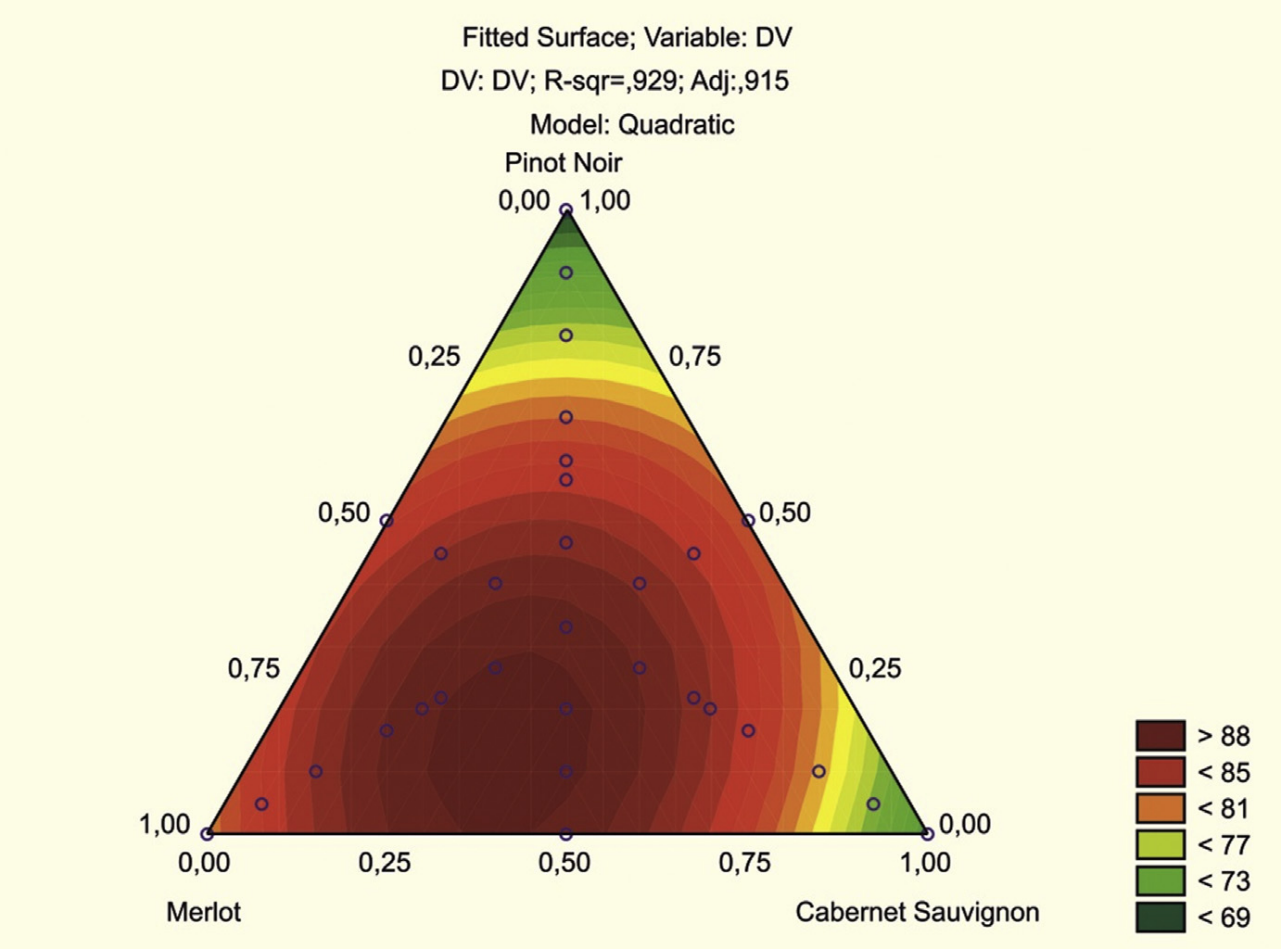
The range of possible values of the blended mixture components.

To better understand the mathematical essence of the method and to illustrate the model results, visualization tools are provided in the module. The restriction of the composition of the mixture components (A + B + C = 100) determines the bounded plane in the three-dimensional space shown in Fig. 1 in the form of a triangle, the apexes of which are named after the components – Merlot, Pinot Noir and Cabernet Sauvignon. It is assumed that the triangle has equal sides of unit length (100%) corresponding to the coordinate axes in the Cartesian coordinate system. The sides of the triangle between the Merlot and Pinot Noir apexes, the Pinot Noir and Cabernet Sauvignon apexes and the Cabernet Sauvignon and Merlot apexes corresponds the respective components of Merlot, Pinot Noir and Cabernet Sauvignon, respectively. The points at the apexes of the mixture correspond to one-component mixtures; those at the sides of the triangle correspond to two-component mixtures (the middles of the sides are mixtures in which both components are present in equal parts); and the points inside the triangle correspond to three-component mixtures. For example, the apex of the Merlot triangle corresponds to Experiment 1, the point in the middle of the Merlot/Pinot Noir side corresponds to Experiment 5, the point inside the triangle closest to the Merlot apex corresponds to Experiment 7, the point inside the triangle closest to the Cabernet Sauvignon apex corresponds to Experiment 8, the point inside the triangle closest to the Pinot Noir apex corresponds to Experiment 9 and the point inside the triangle equidistant from the apexes of the triangle corresponds to Experiment 10.

If you do not select the option augment with interior pts & centroid, then the program will construct a plan consisting of the first 6 experiments that correspond to one-component mixtures at the apex of the triangle — experiments 1–3 and two-component mixtures — experiments 4–6 on the sides of the triangle (Fig. 1). The choice of the option includes in the plan the internal points of the triangle - experiments 7–9; 11–31 and the center point (centroid) - experience 10.

In this way, the plan consisted of 31 experiments, including 3 single-component mixtures, 3 two-component mixtures and 25 three-component mixtures of Merlot, Cabernet Sauvignon and Pinot Noir. Sensory assessment served as a criterion of quality. The average sensory assessments are displayed in the dependent variable (DV) column of the table. As seen, the largest sensory evaluation score (88 points) was achieved by blends 29 and 30 with values of Merlot, Cabernet Sauvignon and Pinot Noir equal respectively to 46.67%, 26.67%, 26.67% and to 26.67%, 26.67, 46.67. But these values were achieved as a result of only 31 experiments from an infinite number of possible mixture combinations.

In fact, using experiment planning means to allow the most equal distribution possible of the points on the triangle to correspond to the experiments, including its sides and apexes. Each of the 31 points on the plane (whose coordinates are the values of the three factors) corresponds to a certain value of the sensory assessment, as given in the last column of Table 1. The greater the value of the sensory assessment is, the darker the coloured area in the corresponding point on the triangle in Fig. 1.

The mathematics of this work consisted of constructing a statistical model based on the results of 31 experiments best expressing the dependence of sensory assessment on the possible mixture combinations. The application of the Analyse design tab options allowed regression models to be constructed in the form of linear and quadratic functions characterizing the relationship between the dependent variable—the sensory assessment—and the values of the wine proportions. In the terminology of regression analysis, sensory assessment (DV) is the response and wine proportions are the predictors. Using ANOVA, the model adequacy was assessed and the optimal proportions of the mixture components were selected. Table 2 shows the indicators characterizing the adequacy of the model: the sum of the squared deviations of the predicted values from their mean value is SS Effect; the same value reduced to the number of degrees (dfEffect) is MS Effect; the sum of the squared deviations of the predicted values from their mean value is SS Error; the same value reduced to the number of degrees of freedom (dfError) is MSError; and the value of the Fisher criterion (F), its significance level (p) and the determination coefficient is *R*^*2*^ (R-sqr). The first four statistics are informative and represent the sum of the squared deviations of the predicted values from their mean value, the same value reduced to the number of freedom degrees (dfEffect) and the sum of the remainders squares (i.e., the difference between the experimental and predicted values). The following three statistics allow us to compare the models. The larger the value of F is, the lower the value of p is and the greater the value of R^2^ is, which means the model more accurately describes the relationship between the response and predictors. As seen, for all three statistics, the quadratic model outperforms the linear model. In addition, the agreement of the adjusted surface is statistically significant for the linear and quadratic model (since p = 0.012 and 0,000, less than 0.05, the accepted level of significance of statistical hypotheses [19]. The determination coefficient R^2^ varies in the range [0, 1] and determines the proportion of the initial variability of the dependent variable relative to the mean value that can be explained by the regression model. Thus, R^2^ = 0.929 means that the model explains approximately 93% of the response variability from the mean value. The linear model determines only about 27% of the variability of the response from the average. Therefore, the optimal composition of the mixture was determined by a quadratic model.

**Table 2:** Dispersion analysis results for the constructed models.

Model	ANOVA; Var.: DV (Star Kuban.sta) 3-factor mixture design; Mixture total = 100, 10 Runs Sequential fit of models of increasing complexity								
	SS Effect	df Effect	MS Effect	SS Error	df Error	MS Error	F	p	R-Sqr
Linear	242.047	2	121.023	656.663	28	23.452	5.160	0.012	0.269
Quadratic	593.068	3	197.689	63.594	25	2.544	77.715	0.000	0.929

Table 3 shows the letter designations of the mixture components, point estimates of the quadratic regression equation coefficients, standard errors, t-test values (Student's test) with significance levels p, interval estimates of coefficients in the form of 95% of confidence intervals. The components of the mixture and their products are called factors by the STATISTICA program. In the model, all factors except for AC are statistically significant (p is greater than 0.05). The rows of the table corresponding to statistically significant factors are bolded.

**Table 3:** Coefficients of the quadratic regression equation.

Factor	Coeffs (recoded comps); Var.:DV; R-sqr = 0.929; Adj:0.915 (Star Kuban.sta); 3-factor mixture design; Mixture total = 100. 31 Runs DV: DV; MS Residual = 2,543					
	Coeff.	Std.Err.	t (25)	P	-95,% Cnf.Limt	+95,% Cnf.Limt
(A)Merlot	0.803	0.012	68.404	0.000	0.779	0.827
(B)Cabernet Sauvignon	0.711	0.012	60.559	0.000	0.687	0.735
(C)Pinot Noir	0.683	0.012	58.173	0.000	0.659	0.707
AB	0.0048	0.001	8.614	0.000	0.004	0.006
AC	0.0034	0.001	6.137	0.000	0.002	0.005
BC	0.0047	0.001	8.389	0.000	0.004	0.006

Using the values of the coefficients from the first column of the table, it is easy to write the quadratic model to predict the DV values (sensory assessment):

(1) Y=0.803·A+0.711·B+0.683·C+0.0048·AB+0.0034·AC+0.0047·BC,

where Y is the DV (the sensory assessment); A, B and C are the proportions of Merlot, Cabernet Sauvignon and Pinot Noir wines, respectively; and AB, AC and BC are products of the corresponding proportions.

In reference to the proportions of the components in the mixture, the predictors A, B and C must satisfy the following additional conditions:

(2) A+B+C=100;A>0;B>0;C>0

Substituting the values of predictors satisfying condition (2) into Eq. (1), it is easy to calculate the predicted value of the response (sensory assessment). First, we are interested in the optimal predictor values of the model (the values of the components of the mixture) that maximize the response (sensory assessment). Unfortunately, the module does not offer the possibility of finding the optimal solution of problems (1) and (2), but there are procedures that allow us to approximate the optimal solution. Below are some of them.

### 3.2 Finding the approximate optimal mixture components

**Fig. 2:**
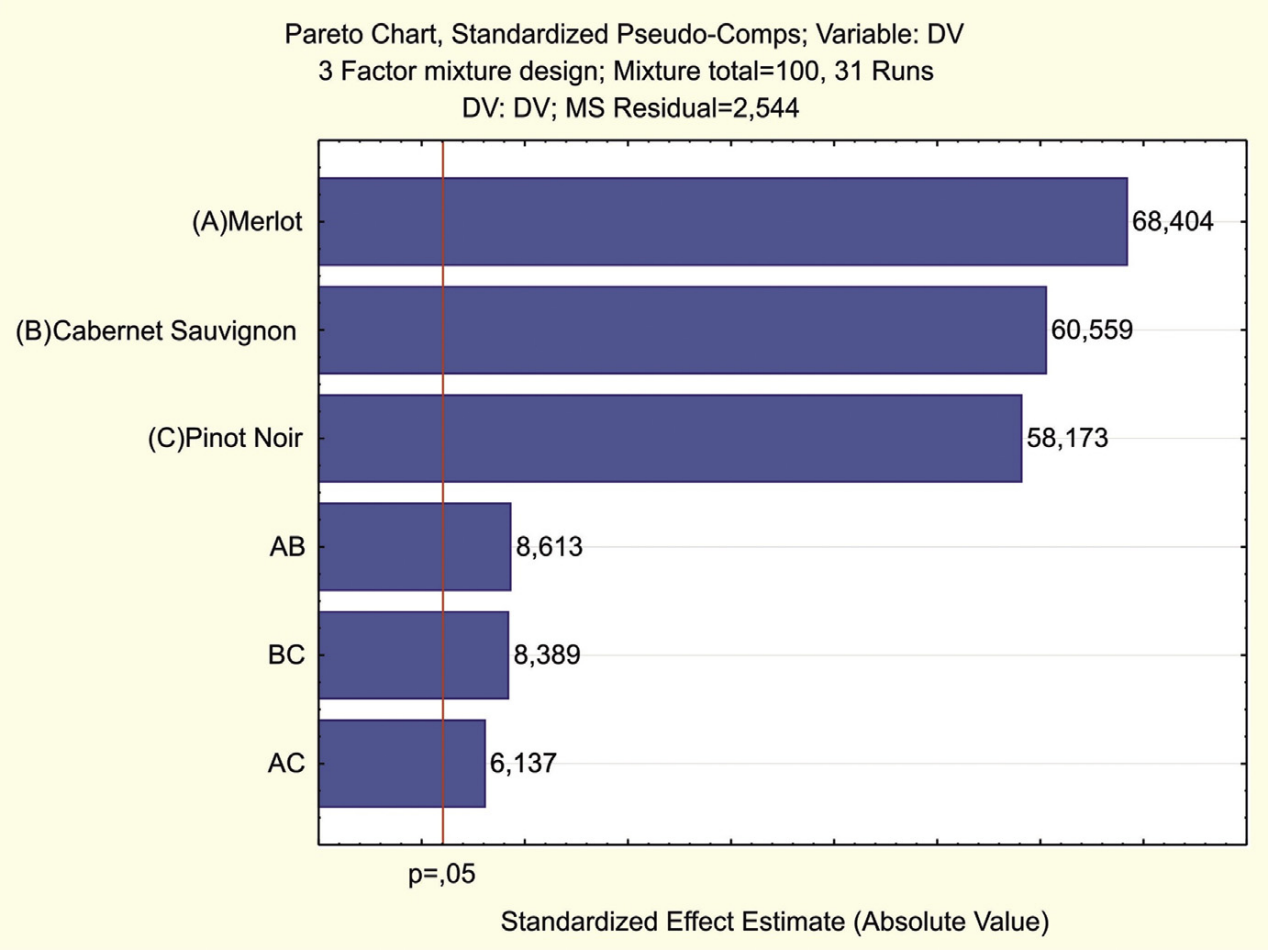
Pareto graph.

One can see the influence of each factor on the response using the quadratic regression coefficient and Student's t-test values; the larger the value is, the higher the contribution to the response. On the Pareto graph (Fig. 2), the model factors are arranged in decreasing order according to their contribution to the response, and the value of Student's t-test is indicated by the vertical line (level of significance p = 0.05). If the column denoting the t-criterion value does not intersect the vertical line, the factor is not statistically significant in the model. The graph shows that the greatest contribution to the value of the sensory evaluation is made by Merlot, followed by Cabernet Sauvignon and Pinot Noir. Assuming the proportions of the mixture components are proportional to the values of the Student's criterion or the equation coefficients, it is easy to calculate the values giving the response an approximately optimal value. Thus, taking a sum of the Student's criterion values 68.404 + 60.559 + 58.173 = 187.136 as 100%, the proportions of Merlot, Cabernet Sauvignon and Pinot Noir can be calculated as 36.55%, 32.36% and 31.09%, respectively.

Using the module options for the found component proportions, the predicted value of the response is easily found. Table 4 shows the results calculated by the program: the predicted sensory assessment point Y is equal to 87.812 (≈88 points) and the interval estimation using the 95% confidence interval is 86.965–88.658. Naturally, the found mixture composition is far from optimal because of the assumption that the optimal mixture of the components corresponds to the contribution of the corresponding factors to the mathematical model.

**Table 4:** Predicted response (sensory assessment) according to factor contributions.

Factor	Predicted Value; Var: DV; R-sqr = 0.929; Adj: 0.915 (Star Kuban.sta); DV: DV; MS Residual = 11.174			
	Coeff.	Pseudo Comp. Val	Coeff.∗ Value	Original Comp. Val
(A) Merlot	80.315	0.365	29.355	36.550
(B) Cabernet Sauvignon	71.105	0.324	23.010	32.360
(C) Pinot Noir	68.303	0.311	21.235	31.090
AB	47.815	0.118	5.655	
AC	34.067	0.114	3.871	
BC	46.565	0.101	4.685	
Predicted			87.812	
-95,% Conf.			86.965	
+95,% Conf.			88.658	

∗ The table shows the coefficients of the pseudo component, provided that their sum = 1.

**Fig. 3:**
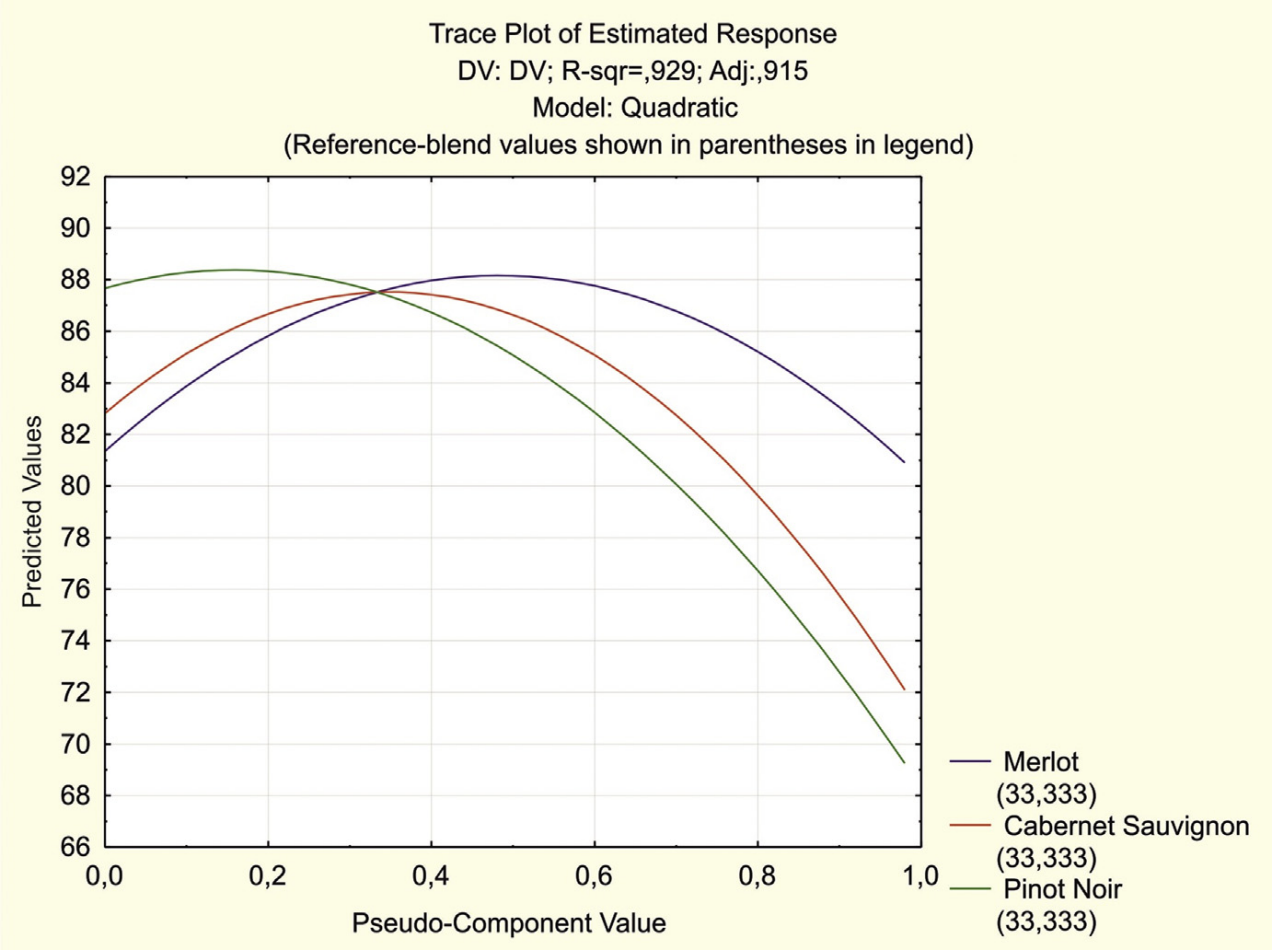
Trace graph of the expected responses (sensory assessments).

Another alternative procedure to find the approximate optimal mixture composition is using trace graphs of the expected responses. When constructing trace curves for each component, all the others are considered basic in the mixture (their proportions remain unchanged) and the proportion of the selected component varies from 0 to 100 %. For each combination, the values of the response function are calculated. The dependence of the given component proportion and the response is presented as a graph, called a trace graph, of the expected values. Fig. 3 shows the expected response curves for each component variation.

Plotting the values of the points on the curves allowed us to determine that the largest predicted values of the sensory assessment are reached if Merlot, Cabernet Sauvignon and Pinot Noir proportions are equal to 48 %, 35 % and 17 %, respectively. With the specified ratios of the components, the predicted value of the sensory assessment slightly increased and reached a value of 88.633 (≈89 points).

**Fig. 4:**
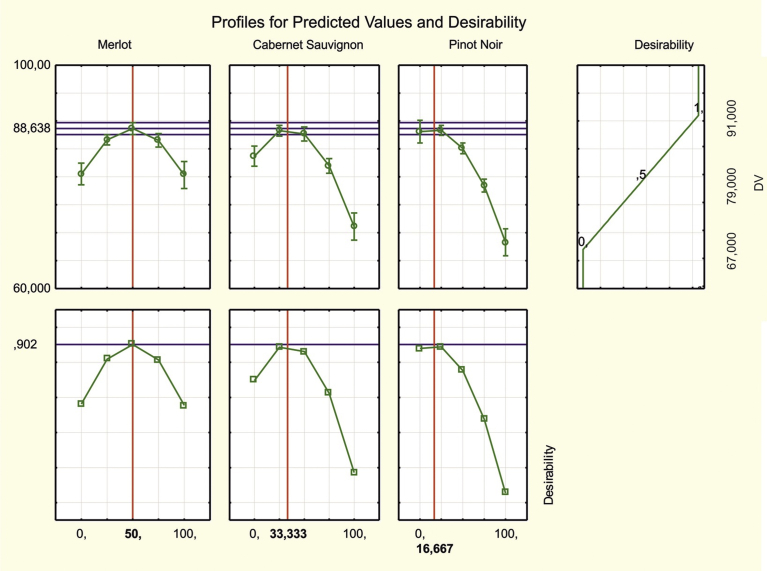
Profiles of predicted values and desirability functions.

You can also find the optimal solution using the command Response desirability profiling, which allows you to view the predicted values for the dependent variables at different combinations of levels of the independent variables, to specify desirability functions for the dependent variables, and to specify a search for the levels of the independent variables that produce the most desirable response on the dependent variables [19]. In Fig. 4 shows the behaviour of the sensory assessment (above) and the desirability function (bottom) with changes in mixture component proportion.

The desirability function within the range from 0 to 1 estimates the degree of preference for the predicted response value with the corresponding value of the given factor and the average values of the remaining factors. The horizontal dashed line on the response graphs shows the highest tasting assessment value achieved by this method (88.638). The horizontal line on the desirability graphs indicates the highest achieved desirability (0.901). The vertical lines correspond to the approximate optimal values of the factors (i.e., the mixture component at which the highest value of the tasting assessment and desirability is achieved): 50% Merlot, 33.33% Cabernet Sauvignon and 16.67% Pinot Noir. At the specified component ratios, the predicted value of the sensory assessment was 88.638 (≈89 points).

Thus, the last two methods gave nearly the same values for the sensory assessment (89 points) with a slight difference in the mixture composition. When making a blend, either of the two obtained solutions can be used, but from the practical point of view, a composition of 48% Merlot, 35% Cabernet Sauvignon and 17% Pinot Noir seems more appropriate.

To check whether the found composition of the mixture and the sensory assessment correspond to the optimal compositions, quadratic programs (1) and (2) were solved using MATLAB [21]. The following values were obtained: Y = 88.699, A = 50.549, B = 35.233 and C = 14.218. The approximate values obtained in the mixture designs and triangular surfaces module differ slightly from the optimal value of the sensory assessment.

**Fig. 5:**
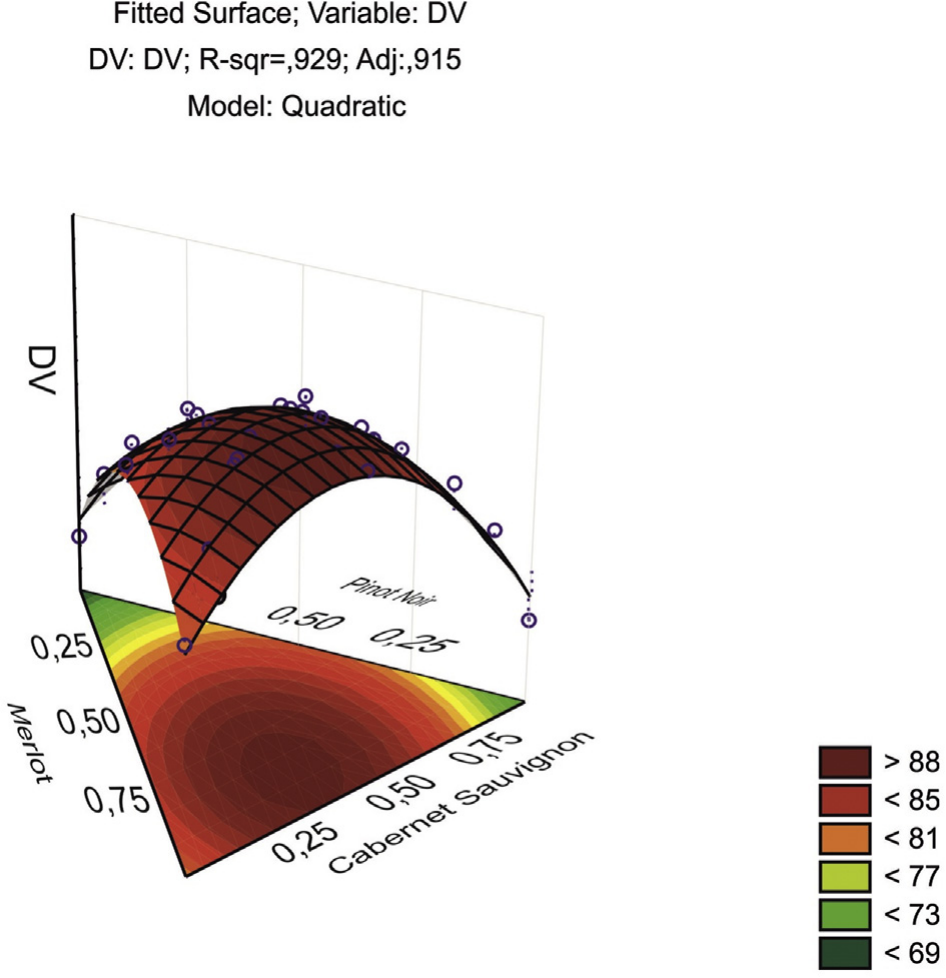
The surface graph fitted by the quadratic function.

In Fig. 5 the quadratic function (1) is shown as a parabolic surface, fitted according to the results of the surface experiments. The experimental values of DV – the sensory assessment – re located either above the surface or below it, but may also appear on the surface itself. The top of this surface – the highest point – corresponds to the mathematically optimal value of the sensory assessment, and its coordinates on the triangle are the mixture component proportions maximizing the quadratic function.

The mixture designs and triangular surfaces module could be used to develop a more complex plan for experiments by considering the limitations of the mixture component composition, as was done by [4], who also used experimental planning in a statistical package environment (SPSS). But, the authors examined 3 responses: the sum of the polyphenols and the flavour and taste preferences of customers. Since we determined the mixture composition only by sensory assessment results, it was considered unnecessary to impose restrictions on the blend components, relying entirely on the experience and qualification of the tasters.

## 4 Conclusions

This paper studied possible mixture designs and the triangular surface method in the STATISTICA package to make blends based on varietal red wines. A mathematically grounded formulation for the new blended wine *Zvezda Kubani* was obtained. It has shown that, to achieve a high sensory assessment, the proportions of the varietal wines Merlot, Cabernet Sauvignon and Pinot Noir in the blend should be 48%, 35% and 17%, respectively. The experimental test of the blended mixture of a predicted composition showed the best values of sensory assessments by the experts. The three-factorial plan consisting of 31 experiments with the three basic varietal wines was used in the work. In the mixture designs and triangular surfaces module of the STATISTICA package, the number of factors can reach 16, and in the design of the constrained surfaces and mixtures module, it can reach 62. Naturally, when experiments are "manually" planned, work with so many factors lacks practical sense.

The use of computer analysis methods can increase the specialists' potential to create varietal food products because they can use not only sensory characteristics but also instrumentally measured quality indicators.

## Declarations

### Author contribution statement

A.A. Khalafyan: Analyzed and interpreted the data.

Z. A. Temerdashev: Conceived and designed the experiments; Analyzed and interpreted the data; Contributed reagents, materials, analysis tools or data; Wrote the paper.

Yu. F. Yakuba: Performed the experiments.

T.I. Guguchkina: Conceived and designed the experiments.

### Funding statement

This work was supported by the Ministry of Education and Science of the Russian Federation, project no. 4.2612.2017/PCh and the Russian Foundation for Basic Research (grant no. 18-03-00059). Experiments were carried out with the use of scientific equipment of the Ecological and Analytical Center of the Kuban State University (unique identifier RFMEFI59317Х0008).

### Competing interest statement

The authors declare no conflict of interest.

### Additional information

No additional information is available for this paper.
